# The Interplay between Conventional Cardiovascular Risk Factors and Health-Related Quality of Life in a Cohort of Working Young and Middle-Aged Adults: A Prospective Study

**DOI:** 10.3390/life12122132

**Published:** 2022-12-16

**Authors:** Cristina Florescu, Petre Ciobanu, Diana Ruxandra Hădăreanu, Veronica Gheorman, Edme Roxana Mustafa, Adina Dorina Glodeanu, Marius Gabriel Bunescu, Adrian Mită, Venera Cristina Dinescu

**Affiliations:** 1 Department of Cardiology, Faculty of Medicine, University of Medicine and Pharmacy of Craiova, 2 Petru Rares St., 200349 Craiova, Romania; 2 Filantropia Clinical Hospital, 200516 Craiova, Romania; 3 Clinical Emergency County Hospital of Craiova, 1 Tabaci St., 200642 Craiova, Romania; 4 Department of Internal Medicine, Faculty of Dentistry, University of Medicine and Pharmacy of Craiova, 2 Petru Rares St., 200349 Craiova, Romania; 5 Department of Health Promotion and Occupational Medicine, Faculty of Medicine, University of Medicine and Pharmacy of Craiova, 2 Petru Rares St., 200349 Craiova, Romania; 6 Department of Medical Semiology, Faculty of Medicine, University of Medicine and Pharmacy of Craiova, 2 Petru Rares St., 200349 Craiova, Romania

**Keywords:** cardiovascular risk factors, cardiovascular diseases, health-related quality of life, interdisciplinarity team, public health

## Abstract

Psychosocial and work stress, socioeconomic status, and environmental health directly impact the onset and progression of cardiovascular diseases, irrespective of sex or conventional cardiovascular risk factors (cCRFs). On the other hand, the impact of cCRFs on health-related quality of life (HRQoL) is not well known, and the psychological socioeconomic environmental somatic health interaction is often neglected. Accordingly, we aimed to: (i) compare the self-reported HRQoL using the WHOQOL-BREF questionnaire between healthy subjects and those with cCRFs; and (ii) evaluate the interplay between HRQoL, cCRFs, and cardiovascular treatment adherence. We prospectively included 90 working adults (46 healthy and 44 with cCRFs age- and sex-matched adults) evaluated by clinical examination, 12-leads electrocardiography, and transthoracic echocardiography as part of a cardiovascular diseases screening program, that also filled in the WHOQOL-BREF questionnaire. Subjects with CRFs were less satisfied with their own individual health. The presence and the number of CRFs, as well as the need for cardiovascular treatment and the number of drugs taken correlated with reduced scores at the majority of HRQoL domains. The results in the social relationships domain were the best predictor of cardiovascular treatment adherence. Finally, the results of all HRQoL domains were strongly correlated to each other demonstrating the psychological socioeconomic environmental somatic health interaction.

## 1. Introduction

Despite significant progress made in the prevention strategies and treatment of cardiovascular (CV) diseases (CVD), they still play the main role in patients’ morbidity and mortality due to chronic diseases worldwide [1]. Furthermore, poor adherence to treatment also contributes to negative outcomes and represents a major health problem [2,3]. While the traditional risk factors of CVDs represented by arterial hypertension, hypercholesterolemia, obesity, Diabetes Mellitus, and tobacco-use are widely recognized, the contribution of psychological, socio-economic factors and environmental factors is often overseen [4,5].

Psychosocial stress is one of the potential CV risk modifiers, that has a direct biological effect on the onset and progression of atherosclerotic CVD [1,6]. It is independently associated with CV health, irrespective of sex and conventional risk factors [7,8], and with all-cause mortality in a large, multiethnic prospective study [9]. Chronic stress leads to arterial hypertension and increased adiposity [10,11], a higher risk of developing Diabetes Mellitus [12], as well as to a higher smoking rate [13]. Low socioeconomic status and work stress are also associated with both psychosocial stress and the development of CVD [8,14]. Similarly, environmental health impacts CV health. Furthermore, the 2021 ESC Guidelines on CVD prevention in clinical practice recommend screening for mental disorders at every consultation in patients with CVD, as well as screening for psychological stress in patients with atherosclerotic CVD [15], because of their impact on CV health and patient prognosis [16,17]. However, despite the evidence underlying the association between the aforementioned factors and CV health, the psychological socioeconomic environmental somatic health interaction is often neglected, and none of these factors have led to a demonstrated improvement in patient risk reclassification [15].

On the other hand, several factors significantly impact health-related quality of life (HRQoL), which is a representation of the impact of health status on everyday life. These factors, apart from the individual health of each person, also include the age, education, marital or socio-economic status, individual circumstances, concerns, goals, as well as the perception of their own health and well-being. The same factors similarly influence patient adherence to medication, which is defined by the World Health Organization as ‘the extent to which a person’s behavior corresponds with agreed recommendations from a health care provider’ [3]. Accordingly, although several subjective, objective or biochemical measurements have been used to assess medication adherence, its evaluation remains challenging because of the individual person’s behavior [18]. Furthermore, despite the fact that poor treatment adherence is reported in 50% of patients with chronic diseases [3], interventions aimed to improve patient adherence to medication showed limited effectiveness because of the multiple reasons for non-adherence [19]. The different aspects of HRQoL might significantly impact treatment adherence. However, the exact relative impact of conventional CV risk factors (CRFs) on the HRQoL, and the relationship between HRQoL and the adherence to the treatment of conventional CRFs are not well understood.

Accordingly, the aims of our study were to: (i) compare the self-reported HRQoL in all four health domains (physical, psychological, social relationships, and environmental) measured using the World Health Organization Quality of Life Brief Version (WHOQOL-BREF) questionnaire between age- and sex-matched healthy subjects and subjects with conventional CRFs participating in a CVD screening program; (ii) evaluate the interplay between conventional CRFs and the results of each of the HRQoL domains; and (iii) evaluate the correlations between the self-reported HRQoL and CV treatment adherence assessed by the Morisky, Green and Levine medication adherence scale (MGLS) [20].

## 2. Materials and Methods

### 2.1. Study Design

**Figure 1:**
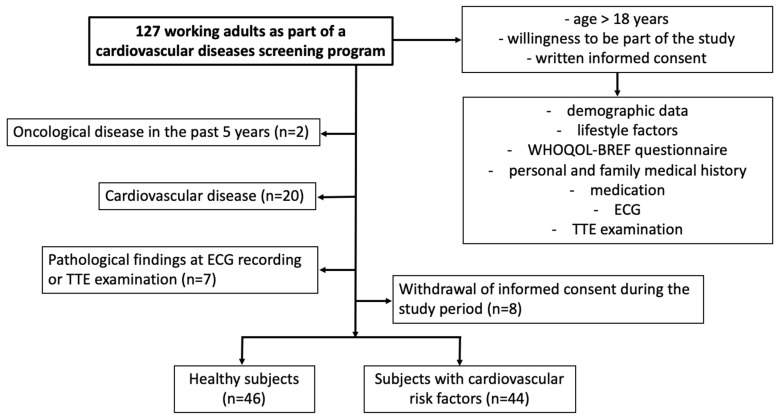
Flowchart describing subjects’ selection process. ECG, electrocardiography; TTE, transthoracic echocardiography.

To fulfill our aims, we prospectively evaluated 127 working young and middle-aged adults from a single Romanian logistics and distribution enterprise between June and October 2022 as part of a CVDs screening program. The inclusion criteria were (i) age ≥18 years, (ii) willingness to be part of the study, and (iii) written informed consent. Exclusion criteria were (i) subjects with clinically-manifested CVD defined as chronic heart failure, cardiomyopathies, chronic coronary syndromes, cerebrovascular disease, prosthetic heart valves, aortic and/or mitral stenosis, and more than mild valvular regurgitation, sustained tachyarrhythmias or bradyarrhythmias, or an acute CV event requiring hospitalization in the past 8 weeks, (ii) oncological diseases in the past 5 years, (iii) pathological findings on either the 12-leads electrocardiographic (ECG) recording or transthoracic echocardiographic (TTE) examination, and (iv) withdrawal of informed consent during the study period. The flowchart describing the subjects’ selection process is shown in Figure 1. Written informed consent was obtained from each participant, and the study was approved by the Ethics Committee of the University of Medicine and Pharmacy of Craiova (No. 166/25.08.2022).

### 2.2. Data Collection

At the time of enrollment in the study, demographical and clinical data including sex, age, body-mass index (BMI), the presence of CRFs, and personal and family medical history were collected for each participant. The CRFs were defined as: (1) current or past history of smoking, (2) dyslipidemia (LDL-Cholesterol > 130 mg/dL or taking lipid-lowering therapies, (3) arterial hypertension (blood pressure ≥ 140/90 mmHg or taking antihypertensive drugs), (4) type 2 Diabetes Mellitus, (5) overweight (BMI = 25–29.9 kg/m^2^) or obesity (BMI ≥ 30 kg/m^2^), and (6) chronic kidney disease [15].

Furthermore, all subjects enrolled in the study underwent a complete physical examination, 12-leads ECG recording, and comprehensive two-dimensional (2D), Doppler, and M-mode transthoracic echocardiography (TTE) performed and interpreted by two experienced cardiologists. They were also asked to fill in the WHOQOL-BREF questionnaire used to assess the self-reported HRQoL [21].

### 2.3. Transthoracic Echocardiography Acquisition and Analysis

The TTE evaluation of the study population was performed using a commercially-available SonoScape S20 ultrasound system (SonoScape Co., Shenzhen, China) equipped with a 2.5 MHz transducer. All echocardiographic measurements were performed as recommended by current guidelines on chamber quantification [22].

Left ventricular (LV) systolic function was evaluated by the ejection fraction (EF) obtained using Simpson’s biplane method of disks summation, and care was taken to avoid LV foreshortening in any of the 2 apical views. LV diastolic function was assessed using the algorithm recommended by the updated guidelines on the evaluation of LV diastolic function by echocardiography [23]. Right ventricular (RV) systolic longitudinal function was determined using the S’ wave velocity at the level of the lateral tricuspid annulus (TA) derived from tissue Doppler imaging and TA plane systolic excursion (TAPSE) derived from M-mode echocardiography [22].

The systolic pulmonary artery pressure (sPAP) was calculated using the maximum velocity of the continuous-wave Doppler jet of the tricuspid regurgitation (TR), and the estimated right atrial pressure based on the size and inspiratory collapsibility of the inferior vena cava [24]. The absence of more than mild valvular regurgitation was confirmed using a multi-parametric algorithm based on 2D and Doppler echocardiographic assessment [25].

### 2.4. Health-Related Quality of Life Evaluation

The WHOQOL-BREF questionnaire used for the self-reported assessment of HRQoL of the participants in the study is one of the most frequently used instruments for measuring HRQoL [26]. It includes 26 questions, of which 24 are divided into 4 domains—physical health, psychological health, social relationships, and environmental health. The remaining 2 questions evaluate the overall perception of the quality of life (QoL) and of individual health, respectively [21].

### 2.5. Medication Adherence Assessment

The self-reported MGLS designed in 1986 [20] used for measuring treatment adherence in our study is still the most widespread questionnaire and provides validity and reliability on several chronic diseases [27]. It comprises four questions with a dichotomous answer of yes or no regarding either (1) the forgetfulness or (2) the carelessness of the patient when taking the medication, and if the patient stops taking the medication in case he/she feels (3) better or (4) worse during the treatment. Answering yes to any of the four questions was considered treatment non-adherence [28].

### 2.6. Statistical Analysis

The distribution of the variables was checked using the Kolmogorov-Smirnov test. Continuous variables are expressed as median and interquartile range because of their skewed distribution, and categorical variables as count and percentage. For intergroup comparison, the Wilcoxon rank-sum statistics were used. For bivariate analyses, the non-parametric Kendall’s tau correlation coefficient and regression analysis were used. A model of multiple regression analysis was computed to assess the interaction between each of the WHOQOL-BREF domains and the other variables used in this study. Receiver-operator characteristics (ROC) curves were derived to compare the capacity of the scores derived from the WHOQOL-BREF questionnaire to predict the subject’s adherence to the cardiological treatment measured by the MGLS. A *p*-value < 0.05 was considered statistically significant. The statistical analysis was performed using SPSS version 23 for Mac (SPSS Inc., IBM Corp., Chicago, IL, USA).

## 3. Results

The final study population consisted of 90 subjects, 46 (51.1%) healthy adults (21 men, median age 37 years) and 44 (48.9%) adults (19 men, median age 45.5 years) with at least one CRF. The demographic, clinical, and paraclinical data of the entire study population and the two subgroups dichotomized based on the presence or absence of CRFs are summarized in Table 1. All subjects included in the study completed tertiary education, and the majority of them (89%) were married or living as married (80/90 subjects).

In the CRFs group, 23 subjects (52.3%) had more than one CRF. Arterial hypertension was present in 18 (40.9%) subjects, 6 (13.6%) were diabetic, 17 (38.6%) were dyslipidemic, 20 (45.5%) were smokers, 21 (47.7%) had a BMI > 25 kg/m^2^, and 0 (0%) had chronic kidney disease. Adherence to treatment was reported by 69 (78.4%) participants in the study.

### 3.1. Comparison between Subjects with and without Cardiovascular Risk Factors

#### 3.1.1. Clinical and Paraclinical Data

There were no statistically significant differences in age and sex distribution between the two groups (*p* > 0.05 for both). As expected, subjects with CRFs were more sedentary (*p* = 0.001) and had higher BMIs (*p* < 0.001) than healthy subjects (Table 1). All subjects were in sinus rhythm on the 12-leads ECG recording. Furthermore, no statistically significant differences were found in terms of heart rate, blood pressure, and TTE parameters between the two groups (*p* > 0.05 for all).

#### 3.1.2. Quality of Life Results

**Figure 2:**
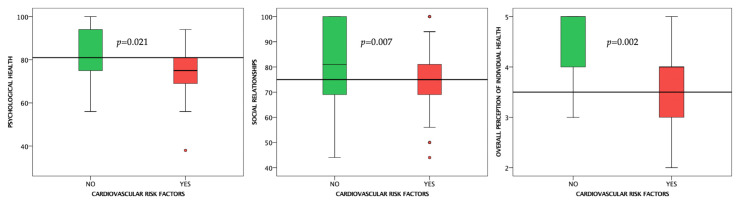
Boxplot charts showing the differences in self-reported HRQoL between subjects with and without cardiovascular risk factors. Horizontal lines are fitted at median.

Subjects in the CRFs subgroup had lower scores at all four domains of the WHOQOL-BREF questionnaire; however, the scores were significantly lower only for the psychological health (*p* = 0.021) and social relationships (*p* = 0.007) domains. Furthermore, the overall perception of the QoL was similar between healthy subjects and those with CRFs (*p* > 0.05). Conversely, the overall perception of individual health was significantly lower in subjects with CRFs (*p* = 0.002) (Figure 2 and Table 2).

### 3.2. Associations between Clinical, Paraclinical and QOL Data

#### 3.2.1. Sedentary Behavior

**Figure 3:**
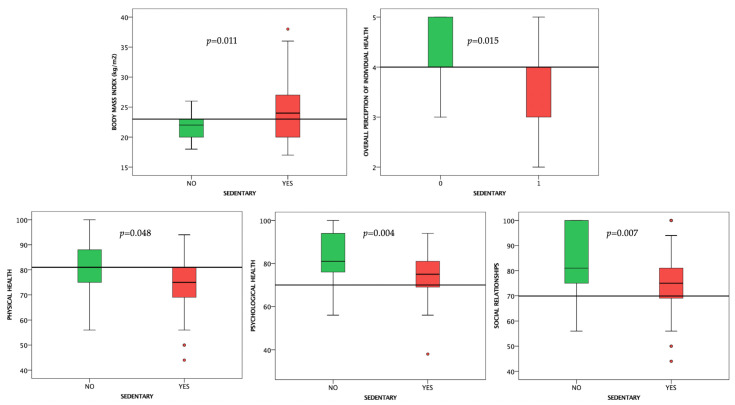
Boxplot charts showing the differences in BMI and WHOQOL-BREF scores between sedentary subjects and subjects that exercised at least once a week. Horizontal lines are fitted at median.

Subjects that were sedentary had higher BMIs (*p* = 0.011), lower results at the physical (*p* = 0.048), psychological (*p* = 0.004), social relationships (*p* = 0.007), and overall perception of individual health (*p* = 0.015) domains compared to subjects that exercised at least once a week (Figure 3).

#### 3.2.2. Arterial Hypertension

**Figure 4:**
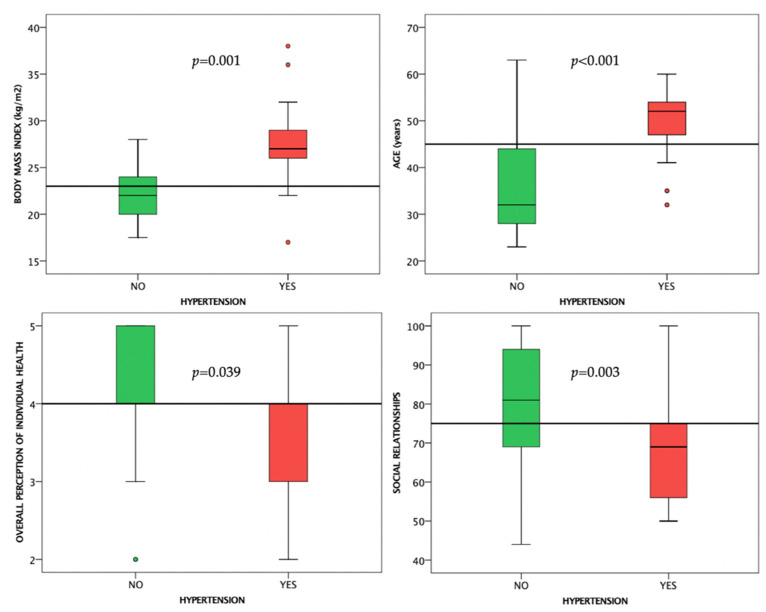
Boxplot charts showing the differences between normotensive and hypertensive subjects. Horizontal lines are fitted at median.

Hypertensive subjects had higher BMIs (*p* = 0.001), were older (*p* < 0.001), and had decreased results in the social relationships WHOQOL-BREF domain (*p* = 0.003), and overall perception of individual health (*p* = 0.039) compared to normotensive subjects (Figure 4).

#### 3.2.3. Type 2 Diabetes Mellitus

There were no differences between diabetic and normoglycemic subjects (*p* > 0.05 for all variables).

#### 3.2.4. Dyslipidemia

**Figure 5:**
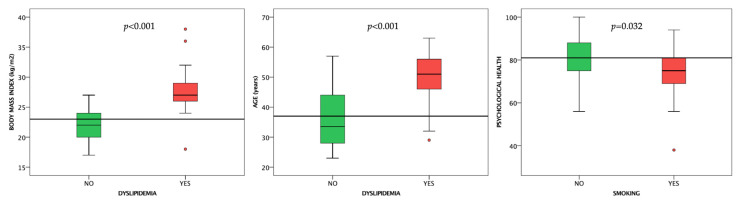
Boxplot charts showing the differences between subjects with and without dyslipidemia, as well as smokers and non-smokers. Horizontal lines are fitted at median.

Dyslipidemic subjects had higher BMIs and were older (*p* < 0.001 for both) than subjects without high serum cholesterol levels (or not taking lipid-lowering drugs) as expected (Figure 5).

#### 3.2.5. Smoking

Current or past smokers had lower psychological health compared to non-smokers (*p* = 0.032) (Figure 5).

#### 3.2.6. Overweight/Obesity

**Figure 6:**
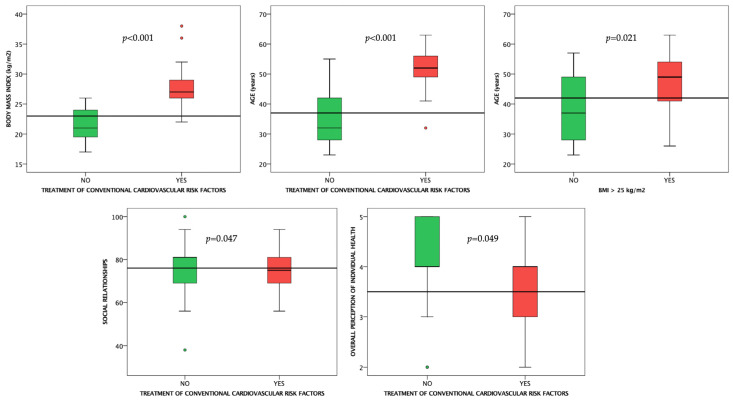
Boxplot charts showing the differences between subjects taking treatment for conventional cardiovascular risk factors and those without, as well as between overweight/obese subjects and those with a BMI ≤ 25 kg/m^2^. Horizontal lines are fitted at median.

Overweight or obese subjects were older than those with BMI < 25 kg/m^2^ (*p* = 0.021, Figure 6).

#### 3.2.7. Treatment of Conventional Cardiovascular Risk Factors

Subjects taking cardiology medication had higher BMIs, were older (*p* < 0.001 for both), and had lower values in the social relationships (*p* = 0.014), and overall perception of individual health (*p* = 0.049) domains compared to subjects not taking any medication (Figure 6). However, there were no statistically significant differences in terms of treatment adherence between any of the subgroups dichotomized based on the specific CRF (*p* > 0.05 for all).

### 3.3. Correlations between WHOQOL-BREF Results and Clinical and Paraclinical Data

#### 3.3.1. WHOQOL-BREF Domains

The results at all four WHOQOL-BREF domains, the overall perception of the individual QoL, and the overall satisfaction with own health were correlated to each other (Table 3), demonstrating the crucial interaction between physical, psychological, social, and environmental health. These findings were also confirmed by bivariate regression analysis (Table 4).

#### 3.3.2. Age, BMI, and the Presence and Number of Cardiovascular Risk Factors

Increased age (r = −0.176, *p* = 0.026) was correlated to lower scores in the physical health domain, while higher BMI to lower scores in the physical health (r = −0.260, *p* = 0.001), social relationships (r = −0.175, *p* = 0.030) and overall perception of individual health (r = −0.185, *p* = 0.033) domains.

The presence and higher number of CRFs were correlated to lower scores at the physical (r = −0.188, *p* = 0.046, and r = −0.259, *p* = 0.003), psychological (r = −0.267, *p* = 0.005 and r = −0.284, *p* = 0.001), social relationships (r = −0.205, *p* = 0.031 and r = −0.235, *p* = 0.008), the overall perception of QoL (r = −0.225, *p* = 0.029 and r = −0.221, *p* = 0.021), and of individual health (r = −0.437, *p* < 0.001 and r = −0.405, *p* < 0.001) domains.

#### 3.3.3. Sedentary Behavior, Type 2 Diabetes Mellitus, Arterial Hypertension, Dyslipidemia, Smoking

Sedentary behavior was correlated to lower scores at the physical (r = −0.272, *p* = 0.004), psychological (r = −0.337, *p* < 0.001), social relationships (r = −0.268, *p* = 0.005), the overall perception of QoL (r = −0.285, *p* = 0.006), and of individual health (r = −0.339, *p* = 0.001) domains.

The presence of type 2 Diabetes Mellitus (r = −0.191, *p* = 0.043), and dyslipidemia (r = −0.314, *p* = 0.001) were correlated with lower physical health results and lower overall perception of individual health (r = −0.248, *p* = 0.015 for both), while arterial hypertension was correlated with lower physical health (r = −0.255, *p* = 0.007), psychological health (r = −0.273, *p* = 0.004), social relationships (r = −0.332, *p* < 0.001), and overall perception of individual health (r = −0.334, *p* = 0.001) scores.

Finally, smoking correlated with lower psychological health (r = −0.250, *p* = 0.008), and overall perception of QoL (r = −0.237, *p* = 0.022).

#### 3.3.4. Cardiovascular Medication and Treatment Adherence

The need for treatment of conventional CRFs correlated with lower physical health (r = −0.315, *p* = 0.001), psychological health (r = −0.228, *p* = 0.016), and social relationships (r = −0.254, *p* = 0.008), and lower overall perception of individual health (r = −0.301, *p* = 0.003).

Taking more than one drug correlated with decreased physical health (r = −0.333, *p* = 0.001), social relationships (r = −0.234, *p* = 0.016), and overall perception of individual health (r = −0.290, *p* = 0.005) domains.

Finally, better CV treatment adherence was correlated with decreased age (r = −0.194, *p* = 0.047), lower BMI (r = −0.202, *p* = 0.040), reduced number of CRFs (r = −0.345, *p* = 0.001), reduced number of medication (r = −0.477, *p* < 0.001), and female sex (r = 0.251, *p* = 0.031).

### 3.4. Multiple Regression Analysis of WHOQOL-BREF Domains’ Results

At multiple regression analysis the number of drugs taken and psychological health independently correlated with physical health. Age, social relationships, and physical health are independently correlated with psychological health. Psychological health independently correlated with social relationships, and the presence and number of CRFs, as well as type 2 Diabetes Mellitus, arterial hypertension, and physical health independently correlated with the overall perception of individual health (Table 5).

### 3.5. Treatment Adherence Predictors Based on WHOQOL-BREF Results

**Figure 7:**
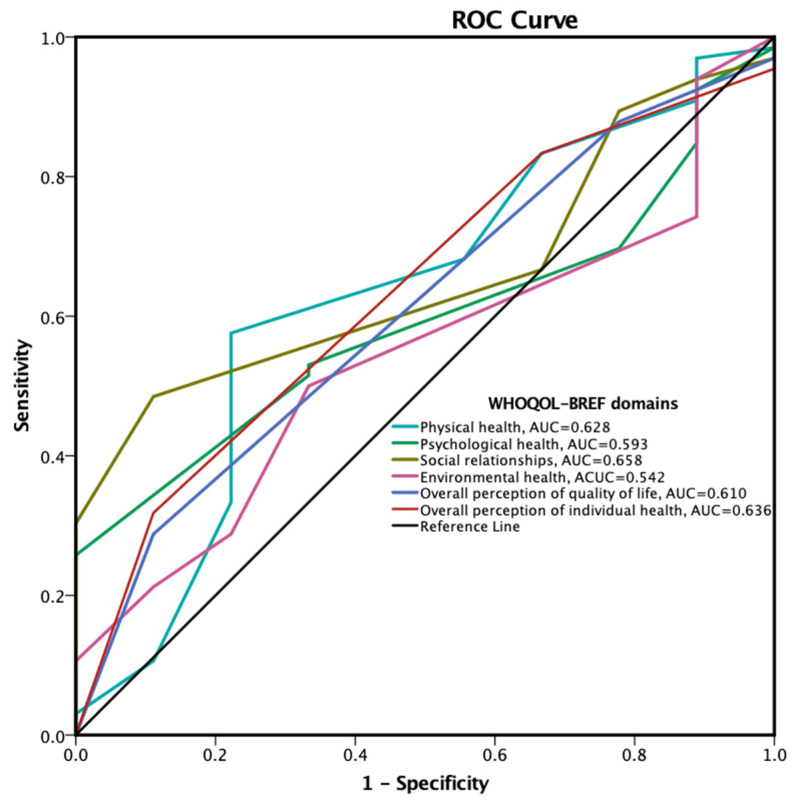
Receiver-operator characteristics curve for the prediction of treatment adherence based on WHOQOL-BREF results. AUC, area under the curve; ROC, receiver-operator characteristics; WHOQOL-BREF, World Health Organization Quality of Life Brief Version.

At ROC analysis, the higher predictive value for the adherence to the treatment of conventional CRFs was found for the social relationships domain (area under the curve, AUC = 0.658), followed by the overall perception of individual health (AUC = 0.636) and the physical health domain (AUC = 0.628) (Figure 7).

## 4. Discussion

To the best of our knowledge, our study is the first to assess the interplay between the self-reported HRQoL of working young and middle-aged adults and the presence of CRFs as well as the need for CV therapies, by comparing two age- and sex-matched groups of subjects with and without CRFs, with similar education levels and socioeconomic status. The main results of our study can be summarized as follows: (i) the results of all four WHOQOL-BREF domains and the overall perception of individual health and individual QoL categories were correlated to each other; (ii) the presence of CRFs as well as more than one CRF correlated to lower scores at the majority of HRQoL domains; (iii) the need for treatment of conventional CRFs and taking more than one drug also correlated with reduced results at most HRQoL domains; (iv) better treatment adherence correlated with decreased age, lower BMI, female sex, fewer CV factors, and a smaller number of drugs taken daily, and (v) out of all WHOQOL-BREF domains, the higher predictive value of CV treatment adherence was found for the social relationships domain.

The direct relationship between increased psychological stress, poor socioeconomic status and reduced environmental health, and the development and progression of CVDs has been the main focus of CV reduction strategies [15], as well as that of the reduction of the global burden of CVDs [29]. However, the presence of conventional CRFs might have a significant negative impact on the physical, psychological or social QoL of adults, before the development of clinically-manifested CVDs [30].

### 4.1. The Presence and Number of Cardiovascular Risk Factors

In our study, we have demonstrated that subjects with CRFs had reduced self-reported HRQoL in the physical and psychological health and social relationships domains as well as in the overall perception of QoL and of individual health. Furthermore, the presence of more than one CRF correlated with even lower HRQoL. Out of the five conventional CRFs assessed, the presence of arterial hypertension correlated with reduced HRQoL in all the physical health, psychological health, and social relationships domains. Increased BMI correlated with lower scores in the physical health and social relationships domains. The presence of dyslipidemia or type 2 Diabetes Mellitus correlated with reduced HRQoL scores at the physical health domain and smoking with lower psychological health. Sedentary behavior impacts every aspect of the HRQoL except for environmental health.

Our results demonstrating the negative impact of arterial hypertension on the HRQoL are consistent with those of previously conducted studies [31]. Mena-Martin et al. evaluated the HRQoL in a random sample of more than 33,000 individuals, proving that hypertensive patients reported poorer HRQoL on all physical, general, and mental health and vitality compared to normotensive individuals [32]. Hayes et al. demonstrated the relationship between hypertension and lower HRQoL in a large study conducted on 8303 adults, with an impact on both physical and mental health, yet patients that were aware of being hypertensive reported poorer health status compared to previously undiagnosed hypertensive patients [33]. Consequently, the presence of arterial hypertension could impact the HRQoL of patients because of their tendency of adopting a particular behavior pattern in response to the awareness of the chronic nature of the disease and the need for medication, and perhaps not necessarily because of the direct consequences of hypertension.

Similar to our findings, previous studies have demonstrated that obesity is associated with decreased HRQoL, particularly regarding the physical health domain [34,35,36,37,38]. In our study, higher BMI not only correlated with decreased physical health, but also impacted the social relationships domain.

The study of Martinelli et al. evaluated the impact of conventional CRFs on HRQoL using the WHOQOL-BREF questionnaire. Their findings are comparable to our results regarding the impact of sedentary behavior, Diabetes Mellitus, dyslipidemia, and having a BMI ≥ 25 kg/m^2^ on HRQoL, as sedentary behavior was inversely associated with lower scores at the psychological health domain, and Diabetes Mellitus, dyslipidemia and BMI ≥ 25 kg/m^2^ with lower scores at the physical health domain [39]. However, in our study, having a BMI ≥ 25 kg/m^2^ and sedentary behavior influenced more than one HRQoL domain, and we have also shown significant correlations between CRFs and the social relationships domains, which they did not find in their study. The contrasting findings between our studies could be attributed to the differences in the age (older subjects), sex distribution (more women), and also probably cultural differences of the participants in their study. Sedentary behavior, via the HRQoL or by its direct consequences, might contribute to increased CV risk, as a higher 1-year daily mean step count correlates with a decrease in atherosclerotic CVD risk [40].

Finally, all the participants in our study had completed tertiary education. This has probably influenced our results, as the level of education is a robust predictor of overall health [41]. Higher education provides better economic well-being and social relationships, less unhealthy behaviors, and more qualitative healthcare [42]. It also reduces the unemployment rates, and all subjects included in our study were chosen from a cohort of working young- and middle-aged adults. Moreover, the majority of the subjects included in our study were married or living as married, and the impact of having emotional support on the HRQoL or treatment adherence cannot be neglected.

### 4.2. The Need for and the Adherence to Cardiovascular Treatment

In our study, we have demonstrated a decrease in HRQoL in subjects taking treatment for CRFs, with even lower results when needing more than one drug. Similar results are reported by a cross-sectional study on 544 patients, in which the need for polypharmacy remained independently associated with lower values at the physical domain of HRQoL. However, their results regarding the impact of polypharmacy on the mental domain of HRQoL are in contrast with ours showing a significant impact of the number of CV drugs also on psychological health [43]. These differences might be explained by the different questionnaires used for the HRQoL assessment. The results of the study by Vyas et al. are, however, in line with ours, showing that patients treated with cardiometabolic risk factors treated with polypharmacy have lower mean scores at the physical and mental component of the survey used to measure their HRQoL [44].

Furthermore, we have demonstrated that treatment adherence has an inversely proportional relationship with the age of the subjects included. Conversely, in the study by Eghbali et al. drug adherence was higher in older patients [45]. The contradictory results may be attributed to the differences in the demographic characteristics of the study populations, as we have not included subjects older than 65 years of age (only adults of working age), having a median age of 42 [29–50] years, and in their study cohort, the mean age of the patients was 60.5 ± 11.5 years. Regarding the impact of either of the HRQoL domains on treatment adherence, we have not found other similar studies to compare to our findings.

Our data provide evidence that conventional CRFs negatively impact the self-reported HRQoL of young and middle-aged working adults without CVDs on multiple levels compared to age- and sex-matched healthy individuals. The relationship between physical, psychological, social, and environmental health is multidirectional, and clinicians should apply a holistic assessment and management of each patient that includes each of the health domains.

### 4.3. Limitations

We recognize the main limitation of our study to be the relatively limited number of subjects included because we aimed to have a well-defined study cohort, of young and middle-aged working adults without established CVD, and with similar age, sex distribution, degree of education, and socio-economic class between those with and without CRFs. Due to the lack of follow-up data, the prognostic significance of our results remains to be demonstrated. Lastly, we have used a subjective method to assess treatment adherence, and despite the wide use and promising validation and reliability of the MGLS [27], neither MGLS, nor any of the other questionnaires used in the studies can be regarded as a gold standard [46]. Accordingly, our findings might not be extrapolated to populations affected by other conditions since different questionnaires might be more suitable for a given situation.

## 5. Conclusions

Subjects with CRFs have a reduced overall perception of their health. The presence and number of CRFs impact the self-related QoL in the physical and psychological health and social relationships domains. The treatment of conventional CRFs and taking more than one drug also correlated with lower results in the majority of HRQoL domains. The self-reported QoL in the social relationships domain was the best predictor for CV treatment adherence in our study. Finally, all HRQoL domains were correlated to each other, demonstrating the crucial psychological socioeconomic environmental somatic health interaction. More efficient strategies aiming at further reducing the CV risk of subjects with potential risk modifiers remain to be developed based on our results.

## Figures and Tables

**Table 1:** Demographic, clinical, and paraclinical parameters of the study population.

Parameter	All Subjects (*n* = 90)	Healthy Subjects (*n* = 46)	Subjects with Cardiovascular Risk Factors (*n* = 44)	*p* Value
Age (years)	42 [29–50]	37 [28.5–48.5]	45.5 [28.75–52.25]	0.245
Men (%)	33 (44)	21 (45.7)	19 (45.2)	0.713
Body-mass index (kg/m^2^)	23 [20–25.5]	22 [20–23.75]	25.5 [20–27]	<0.001
Sedentary (%)	46 (51.1%)	12 (26.1%)	34 (77.3%)	0.001
Heart rate (bpm)	70 [65–80]	70 [65–77.5]	70 [65.75–78.25]	0.923
Systolic blood pressure (mmHg)	115 [110–120]	120 [110–120]	110 [110–125]	0.1
Diastolic blood pressure (mmHg)	60 [55–70]	67.5 [60–70]	65 [60–70]	0.680
Sinus rhythm (%)	90 (100%)	46 (100%)	44 (100%)	0.989
Biplane LVEF (%)	58 [55–60]	58 [55–60]	57 [55–59]	0.997
M-mode RV TAPSE (mm)	21 [18–25]	21 [19–25]	21 [19–24]	0.999
TDI S’ wave velocity (cm/s)	13 [10–15]	13 [10–16]	12 [9–14]	0.998
E/A ratio	1.5 [1.1–1.7]	1.6 [1.1–1.9]	1.4 [1.1–1.6]	0.879
Average E/e’ ratio	6 [4–7]	5 [4–7]	6 [4–7]	0.991
sPAP (mmHg)	16 [15–22]	17 [14–21]	19 [17–22]	0.993

Continuous variables are expressed as median and interquartile range. Categorical variables are expressed as count and percentage. Abbreviations: A, peak velocity of the pulsed-wave Doppler late diastolic wave at the level of the tips of the mitral valve leaflets; E, peak velocity of the pulsed-wave Doppler early diastolic wave at the level of the tips of the mitral valve leaflets; e’, average tissue Doppler imaging early diastolic wave at the level of the medial and lateral mitral annulus; LVEF, left ventricular ejection fraction; S’, tissue Doppler imaging peak systolic wave at the level of the lateral tricuspid annulus; sPAP, systolic pulmonary artery pressure; TAPSE, tricuspid annular plane systolic excursion; TDI, tissue Doppler imaging.

**Table 2:** Quality of life results of the study population and comparison between subjects without and with conventional cardiovascular risk factors.

WHOQOL-BREF Domain	All Subjects (*n* = 90)	Healthy Subjects (*n* = 46)	Subjects with Cardiovascular Risk Factors (*n* = 44)	*p* Value
Physical health	81 [69–88]	81 [72–88]	75 [63–88]	0.640
Psychological health	81 [69–88]	81 [75–94]	75 [69–84]	0.021
Social relationships	75 [69–94]	81 [69–100]	75 [69–84]	0.007
Environmental health	75 [64–81]	75 [69–84.5]	69 [63–83]	0.497
Overall perception of quality of life				0.240
Very poor	0 (0%)	0 (0%)	0 (0%)	
Poor	3 (3.3%)	0 (0%)	3 (6.8%)	
Neither poor nor good	10 (11.1%)	2 (4.3%)	8 (16.7%)	
Good	50 (55.5%)	27 (58.7%)	23 (54.8%)	
Very good	27 (31.1%)	17 (37%)	10 (23.8%)	
Overall satisfaction with own health				0.002
Very dissatisfied	0 (0%)	0 (0%)	0 (0%)	
Dissatisfied	3 (3.3%)	0 (0%)	3 (6.8%)	
Neither satisfied nor dissatisfied	12 (13.3%)	1 (2.2%)	11 (25%)	
Satisfied	43 (47.8%)	22 (47.8%)	21 (47.7%)	
Very satisfied	32 (35.6%)	23 (50%)	9 (20.5%)	

Variables are expressed as median and interquartile range. Abbreviations: QOL, quality of life; WHOQOL-BREF, World Health Organization Quality of Life Brief Version.

**Table 3:** Kendall tau’s correlations between the results of the WHOQOL-BREF questionnaire domains.

WHOQOL-BREF Domain	Physical Health	Psychological Health	Social Relationships	Environmental Health	Overall QoL	Overall Health
Physical health		r = 0.396	r = 0.228	r = 0.178	r = 0.346	r = 0.435
		*p* < 0.001	*p* = 0.006	*p* < 0.001	*p* < 0.001	*p* < 0.001
Psychological health	r = 0.396		r = 0.435	r = 0.380	r = 0.459	r = 0.489
	*p* < 0.001		*p* < 0.001	*p* < 0.001	*p* < 0.001	*p* < 0.001
Social relationships	r = 0.228	r = 0.435		r = 0.312	r = 0.399	r = 0.357
	*p* = 0.006	*p* < 0.001		*p* < 0.001	*p* < 0.001	*p* = 0.001
Environmental health	r = 0.178	r = 0.380	r = 0.312		r = 0.350	r = 0.273
	*p* < 0.001	*p* < 0.001	*p* < 0.001		*p* < 0.001	*p* = 0.002
Overall QoL	r = 0.346	r = 0.459	r = 0.399	r = 0.350		r = 0.398
	*p* < 0.001	*p* < 0.001	*p* < 0.001	*p* < 0.001		*p* < 0.001
Overall health	r = 0.435	r = 0.489	r = 0.357	r = 0.273	r = 0.398	
	*p* < 0.001	*p* < 0.001	*p* = 0.001	*p* = 0.002	*p* < 0.001	

Kendall tau’s correlation was used at a two-tailed level. Abbreviations: QOL, quality of life; r, Kendall tau’s correlation coefficient; WHOQOL-BREF, World Health Organization Quality of Life Brief Version.

**Table 4:** Bivariate regression analysis between the WHOQOL-BREF domains.

Dependent Variable	Independent Variable	R^2^	Beta	*p*
Physical health	Psychological health	0.328	0.572	<0.001
	Social relationships	0.100	0.317	0.003
	Environmental health	0.063	0.252	0.019
	Overall perception of quality of life	0.235	0.485	<0.001
	Overall perception of individual health	0.293	0.541	<0.001
Psychological health	Social relationships	0.281	0.530	<0.001
	Environmental health	0.240	0.489	<0.001
	Overall perception of quality of life	0.327	0.571	<0.001
	Overall perception of individual health	0.345	0.594	<0.001
Social relationships	Environmental health	0.148	0.384	<0.001
	Overall perception of quality of life	0.156	0.395	<0.001
	Overall perception of individual health	0.175	0.418	<0.001
Environmental health	Overall perception of quality of life	0.156	0.395	<0.001
	Overall perception of individual health	0.106	0.326	0.002
Overall perception of quality of life	Overall perception of individual health	0.244	0.493	<0.001

**Table 5:** Multiple regression analysis models.

Dependent Variable	Independent Variables	Beta	*p*
Physical health	Number of drugs	−0.414	0.022
	Psychological health	0.134	0.004
	Overall perception of individual health	0.333	0.013
Psychological health	Age	0.225	0.048
	Social relationships	0.270	0.009
	Physical health	0.339	0.004
Social relationships	Psychological health	0.430	0.009
Environmental health			>0.05 for all
Overall perception of quality of life			>0.05 for all
Overall perception of individual health	Presence of cardiovascular risk factors	−0.595	0.002
	Number of cardiovascular risk factors	0.802	0.041
	Type 2 Diabetes Mellitus	−0.349	0.010
	Arterial hypertension	−0.442	0.042
	Physical health	0.316	0.013

Each model included as independent variables age, sex, body-mass index, the presence and number of cardiovascular risk factors, the presence or absence of type 2 Diabetes Mellitus, arterial hypertension, dyslipidemia, smoking, overweight/obesity, sedentary behavior, the need for and the number of cardiovascular drugs, and the WHOQOL-BREF domains’ scores. Only the statistically significant correlations were reported in the table.

## Data Availability

The data presented in this study are available on request from the corresponding author, following approval from the University of Medicine and Pharmacy of Craiova, Romania.
